# Sub-maximal endurance exercise does not mediate alterations of somatosensory thresholds

**DOI:** 10.1038/s41598-020-67700-4

**Published:** 2020-07-01

**Authors:** Ann-Christin Kortenjann, Winfried Banzer, Johannes Fleckenstein

**Affiliations:** 1 Department of Sports Medicine, Institute of Sports Sciences, Goethe-University of Frankfurt, Ginnheimer Landstr. 39, 60487 Frankfurt am Main, Germany; 2 Institute of Occupational, Social and Environmental Medicine, Goethe-University, 60590 Frankfurt, Germany

**Keywords:** Sensory processing, Preventive medicine

## Abstract

Physical exercise has been shown to alter sensory functions, such as sensory detection or perceived pain. However, most contributing studies rely on the assessment of single thresholds, and a systematic testing of the sensory system is missing. This randomised, controlled cross-over study aims to determine the sensory phenotype of healthy young participants and to assess if sub-maximal endurance exercise can impact it. We investigated the effects of a single bout of sub-maximal running exercise (30 min at 80% heart rate reserve) compared to a resting control in 20 healthy participants. The sensory profile was assessed applying quantitative sensory testing (QST) according to the protocol of the German Research Network on Neuropathic Pain. QST comprises a broad spectrum of thermal and mechanical detection and pain thresholds. It was applied to the forehead of study participants prior and immediately after the intervention. Time between cross-over sessions was one week. Sub-maximal endurance exercise did not significantly alter thermal or mechanical sensory function (time × group analysis) in terms of detection and pain thresholds. The sensory phenotypes did not indicate any clinically meaningful deviation of sensory function. The alteration of sensory thresholds needs to be carefully interpreted, and only systematic testing allows an improved understanding of mechanism. In this context, sub-maximal endurance exercise is not followed by a change of thermal and mechanical sensory function at the forehead in healthy volunteers.

## Introduction

Exercise-induced changes of the somatosensory profile have led to controversial discussions, in particular with regards to their effect on pain. Exercise has been shown to induce hypoalgesia in healthy subjects^1^, however, it can also induce hyperalgesia in certain chronic diseases or following exertion^2^. Its effects in people with clinical pain are rather variable. In patients with neuropathic pain, exercise is considered to improve nerve function, reduce pain and other sensory dysfunction, and improve static and dynamic functional mobility^3^. However, in patients with musculoskeletal pain, manual therapies on stability failed to achieve a significant pain reduction^4^. A recent systematic review suggests an association between psychosocial factors and exercise-induced hypoalgesia in healthy subjects, and in patients with musculoskeletal pain^5^. This is further supported by a proposed central mechanism underlying these two phenomena, such as the increased activation of NMDA receptors in pain-modulating areas^6^.

Exercise-induced hypoalgesia, i.e. pain reduction, can be achieved by both aerobic and resistance training, of which the latter achieves larger mean effect sizes^7^. Aerobic exercise is more likely to induce generalised hypoalgesia, whereas resistance exercise is related to locoregional effect sites^1^. Higher aerobic intensities (200 W or 60–75% oxygen uptake) appear more effective in mediating hypoalgesia. Isometric exercise was shown to decrease the intensity of experimental pain at loads around 25% of maximum voluntary contraction^8^. Physiologic research points to the activation of the endogenous opioid^9^ and cannabinoid system^10^ as a cardinal mechanism of exercise-induced hypoalgesia.

Exercise-induced hyperalgesia, the phenomenon of increased pain perception following exercise, is more likely to be observed in patients with conditions such as myalgic encephalomyelitis^2^, chronic fatigue syndrome^2,11^, fibromyalgia^12^, or painful diabetic neuropathy^13^. It has been suggested that a different response of the immune system—which is already weakened in certain chronic diseases—partially contributes to this phenomenon. The increased sensitivity to exercise in musculoskeletal pain has also been linked to psychological factors such as catastrophizing and inability to disengage, as well as other processes related to the central sensitisation of pain^14^.

The current literature’s main limitations are of methodologic nature, as many studies fail to adhere to current recommendations on exercise doses, or accurately report the applied exercise loads^1^. In addition, many studies base their findings on the assessment of single sensory thresholds, thereby decoding only parts of the complex physiologic network underlying sensory phenotypes^1,7,15^. Finally, exercise-induced hypoalgesia is widely defined as decreased sensitivity to externally induced painful stimuli^7^, therefore assessing an experimental instead of a clinical setting.

The aim of this present study was to determine the immediate, exercise-induced effects on sensory function. Taking into account the aforementioned observations, we designed our study to allow a systematic description of the pattern of sensory loss and gain caused by a single bout of aerobic exercise, which may reflect the underlying physiology^15^. Quantitative sensory testing (QST) has been established as the clinical standard to assess a multitude of sensory thresholds, by applying predetermined physical stimuli^16^, reliably describing an individual’s sensory profile, and identifying sensory deficits or hyperphenomena.

## Methods

### Study design

A randomised, controlled cross-over study investigating the effects of a vigorous exercise intervention on sensory thresholds in healthy adults. The study has been approved by the Local Ethics Committee FB05 of the Goethe University of Frankfurt, Germany (reference 2017-028) and is in accordance with the Declaration of Helsinki (Version Fortaleza 2012). Inclusion criteria were volunteers between age 18 and 30. Exclusion criteria were pregnancy or lactation, continuous use of analgesics and/or intake within 24 h prior to the study, acute or chronic pain, sensory disorders, and severe illness affecting the quality of life^17^.

Written informed consent was obtained by all participants. After enrolment, participants were subsequently randomised to either Group 1 or Group 2, using an online-based randomisation software stratifying for gender^18^. Groups differed with regards to the sequence of the intervention (vigorous exercise) and control setting:

- Group 1: Assessment–Exercise–Assessment–Wash Out (1 week)–Assessment–Control–Assessment.
- Group 2: Assessment–Control–Assessment–Wash Out (1 week)–Assessment–Exercise–Assessment.

Participants had to perform 30 min of running exercise on a track. The individual intensity was calculated applying the Karvonen formula (80% heart rate reserve) and monitored by a pulse watch (Polar S810®, Kempele, Finland). For the control procedure, participants were sitting for 30 min, with continuous heart rate monitoring to exclude any physical workload.

Sensory thresholds were assessed using the QST method at baseline (i.e. prior to) and immediately following either intervention or control. QST consists of 7 tests measuring 13 parameters in order to assess and quantify the perception of temperature, touch, pain, pressure, and vibration. We could previously demonstrate that the adherence of the proposed test order of QST is relevant in interpreting the obtained results^19^. The detailed QST protocol including reference data is reported elsewhere^16^.

- *Thermal Testing* comprising cold and warm detection thresholds (CDT, WDT), paradoxical heat sensations (PHS) during the thermal sensory limen procedure (TSL) of alternating warm and cold stimuli and cold and heat pain thresholds (CPT, HPT).
- *Mechanical Testing* comprising mechanical detection thresholds (MDT), mechanical pain thresholds (MPT), mechanical pain sensitivity (MPS), dynamic mechanical allodynia (DMA), the wind-up ratio (WUR), vibration detection thresholds (VDT) and pressure pain thresholds (PPT).

QST was performed at the forehead.

### Sample size estimation

The sample size was estimated based on a previous study suggesting an effects size of 0.65 for the change in pressure pain threshold at the forehead^20^. Applying an α of 0.05, and a power of 0.95, we calculated a total sample size of n = 40, taking into account a 20% drop-out ratio. Applying a cross-over study design, n = 20 participants were needed.

### Statistics

QST data were analysed as described by Rolke et al*.*^16^, i.e. some of the data were log-transformed in order to achieve normally distributed data. Consequently, data were compared using a two-way analysis of variance for repeated measures (time × group 2 × 2).We calculated the mean differences for all parameters, which were analysed by paired Students’ *t* test. Finally, for comparisons of the QST data profile following exercise or rest with the baseline data, all parameters were Z-transformed by using the equation:

$$ Zscore = (X_{single\,participant} - Mean _{Baseline } )/SD_{Baseline} $$

The Z-score values were presented with values above “0” indicating a gain of function and below “0” indicating a loss of function^16^.

## Results

Twenty healthy volunteers (female n = 10, age 25 ± 2.3 years, weight 69.8 ± 13.5 kg, height 174.3 ± 9.4 cm) participated in this study, and no drop-outs occurred. Heart rate at rest was 56.65 ± 8.03 min^−1^, and HRR80 was calculated at 160.75 ± 3.34 min^−1^. During exercise, the mean heart rate was 160.19 ± 7.5 min^−1^, and during control (rest) 56.49 ± 7.12 min^−1^. Exercise intensity was rated 12.9 ± 1.74 (“somewhat hard”) on a Borg-scale.

Wash-out between sessions was successful, with equal baseline thresholds prior to both sessions.

Time × group analysis (2 × 2) could not detect any significant differences between groups over time (*p* > 0.05; see Table 1) ^17^.

**Table 1:** Baseline and post-intervention/-control sensory thresholds, indicated as mean ± standard deviation.

	Group	Baseline (Mean ± SD)	Post (Mean ± SD)	Baseline (Mean^log^ ± SD^log^)	Post (Mean^log^ ± SD^log^)	Time × group analysis (*p* value)
*Thermal thresholds*						
CDT (°C)	Intervention	− 0.59 ± 0.49	− 0.95 ± 0.89	− 0.22 ± 0.18	− 0.07 ± 0.26	F_1,38_(0.641) 0.428
	Control	− 1.02 ± 0.65	− 0.83 ± 0.37	− 0.07 ± 0.26	− 0.12 ± 0.19	
WDT (°C)	Intervention	0.73 ± 0.33	1.07 ± 0.82	− 0.17 ± 0.18	− 0.16 ± 0.27	F_1,38_(1.215) 0.251
	Control	1.70 ± 1.07	1.22 ± 0.84	0.16 ± 0.26	− 0.01 ± 0.30	
TSL (°C)	Intervention	1.50 ± 0.95	1.97 ± 0.86	0.18 ± 0.20	0.32 ± 0.24	F_1,38_(0.031) 0.862
	Control	2.43 ± 1.43	1.78 ± 0.83	0.32 ± 0.24	0.21 ± 0.20	
CPT (°C)	Intervention	− 4.07 ± 9.25	− 4.13 ± 8.58			F_1,38_(1.371) 0.2491
	Control	− 5.32 ± 6.24	− 8.12 ± 9.18			
HPT (°C)	Intervention	23.47 ± 12.57	26.30 ± 14.20			F_1,38_(0.015) 0.902
	Control	24.29 ± 11.25	26.23 ± 13.06			
PHS (n/3)	Intervention	0/3	0/3			n/a
	Control	0/3	0/3			
*Mechanical thresholds*						
MDT (mN)	Intervention	0.34 ± 0.27	0.26 ± 0.06	− 0.55 ± 0.25	− 0.47 ± 0.26	F_1,38_(0.364) 0.550
	Control	0.42 ± 0.32	0.32 ± 0.23	− 0.47 ± 0.27	− 0.45 ± 0.49	
MPT (mN)	Intervention	37.44 ± 37.12	42.39 ± 42.72	1.39 ± 0.41	1.32 ± 0.28	F_1,38_(0.137) 0.713
	Control	25.90 ± 17.66	26.77 ± 18.67	1.32 ± 0.28	1.33 ± 0.31	
MPS (0–100)	Intervention	2.97 ± 3.30	2.81 ± 1.94			F_1,38_(0.573) 0.454
	Control	3.66 ± 4.88	3.46 ± 2.41			
Allodynia (0–100)	Intervention	0.00 ± 0.01	0.01 ± 0.03			F_1,38_(0.122) 0.729
	Control	0.00 ± 0.01	0.01 ± 0.02			
WUR (0–10)	Intervention	3.42 ± 3.41	4.34 ± 4.16	0.40 ± 0.32	0.47 ± 0.25	F_1,38_(0.985) 0.327
	Control	3.57 ± 2.70	4.61 ± 3.24	0.48 ± 0.25	0.56 ± 0.31	
VDT (/8)	Intervention	7.07 ± 0.86	6.80 ± 0.94			F_1,38_(0.178) 0.676
	Control	6.97 ± 0.81	7.03 ± 0.80			
PPT (kPa)	Intervention	554.85 ± 154.00	559.70 ± 253.31	2.73 ± 0.12	2.75 ± 0.09	F_1,38_(0.041) 0.841
	Control	574.55 ± 120.94	558.60 ± 251.50	2.75 ± 0.09	2.72 ± 0.16	

Some data are logarithmic, according to the handbook.

*CDT* cold detection threshold, *WDT* warm detection threshold, *TSL* thermal sensory limen, *CPT* cold pain threshold, *HPT* heat pain threshold, *PHS* paradoxical heat sensation, *MDT* mechanical detection threshold, *MPT* mechanical pain threshold, *MPS* mechanical pain sensitivity, *WUR* wind up ratio, *VDT* vibration detection threshold, *PPT* pressure pain threshold.

**Figure 1:**
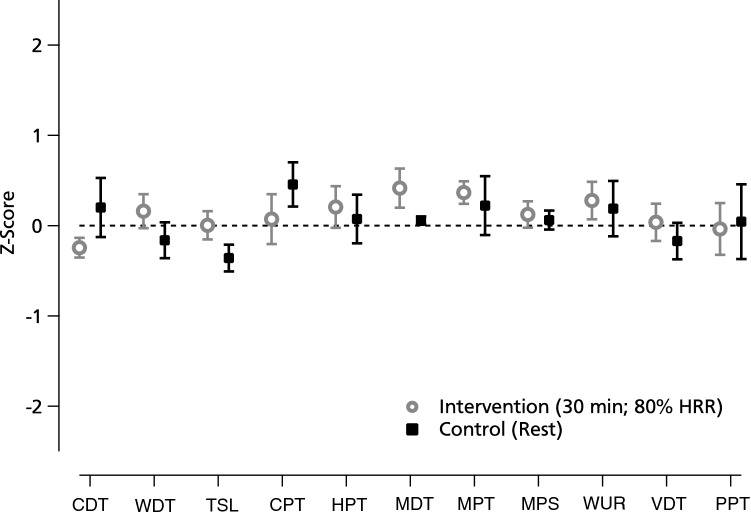
Z-profile of sensory thresholds following intervention (open circles) or control (black squares). Z-score values reflect the patient’s sensitivity for each parameter compared to the group mean at baseline. Z-values above “0” indicate a gain of function when the patient is more sensitive to the tested stimuli compared with controls, while Z-scores below “0” indicate a loss of function referring to a lower sensitivity of the patient^16^. *CDT* cold detection threshold, *WDT* warm detection threshold, *TSL* thermal sensory limen, *CPT* cold pain threshold, *HPT* heat pain threshold, *PHS* paradoxical heat sensation, *MDT* mechanical detection threshold, *MPT* mechanical pain threshold, *MPS* mechanical pain sensitivity, *WUR* wind up ratio, *VDT* vibration detection threshold, *PPT* pressure pain threshold.

There was also no statistically significant difference detected when analysing difference scores (*p* > 0.05; see Table 2)^17^. Z-profiles indicated no differences between groups, nor did we detect deviations larger than the twofold standard deviation within our study group, which would indicate pathologic findings (see Fig. 1).

**Table 2:** Mean differences (baseline to post-intervention/-control) of sensory thresholds.

	Group	(Mean ± SD)	(Mean^log^ ± SD^log^)	Paired *t* test (*p* value)
*Thermal thresholds*				
CDT (°C)	Intervention	0.36 ± 0.91	− 0.65 ± 0.75	0.950
	Control	− 0.19 ± 0.60	− 0.64 ± 0.54	
WDT (°C)	Intervention	− 0.34 ± 0.87	− 0.54 ± 0.75	0.142
	Control	0.48 ± 0.95	− 0.35 ± 0.73	
TSL (°C)	Intervention	− 0.47 ± 1.03	− 0.33 ± 0.60	0.800
	Control	0.66 ± 1.32	− 0.39 ± 0.83	
CPT (°C)	Intervention	− 0.06 ± 8.57		0.134
	Control	− 2.80 ± 9.54		
HPT (°C)	Intervention	− 2.83 ± 18.80		0.439
	Control	− 1.95 ± 15.47		
PHS (n/3)	Intervention	0/3		n/a
	Control	0/3		
*Mechanical thresholds*				
MDT (mN)	Intervention	0.09 ± 0.28	− 1.19 ± 0.67	0.448
	Control	0.09 ± 0.29	− 1.00 ± 0.89	
MPT (mN)	Intervention	− 4.95 ± 57.77	0.79 ± 1.42	0.989
	Control	− 0.87 ± 22.80	0.79 ± 1.05	
MPS (0–100)	Intervention	0.17 ± 3.24		0.947
	Control	0.20 ± 3.91		
Allodynia (0–100)	Intervention	− 0.01 ± 0.03		0.640
	Control	− 0.00 ± 0.02		
WUR (0–10)	Intervention	− 0.92 ± 5.02	0.10 ± 0.64	0.802
	Control	− 1.04 ± 3.19	0.08 ± 0.62	
VDT (/8)	Intervention	0.10 ± 0.75		0.080
	Control	− 0.23 ± 0.82		
PPT (kPa)	Intervention	− 4.85 ± 234.37	1.78 ± 1.20	0.350
	Control	15.95 ± 258.08	2.04 ± 0.41	

Differences have been calculated as baseline minus follow-up, consequently negative differences indicate an increased threshold. In negative variables (i.e. CDT and CPT), negative differences indicate a decreased threshold. Some of the data are logarithmic, according to the handbook.

*CDT* cold detection threshold, *WDT* warm detection threshold, *TSL* thermal sensory limen, *CPT* cold pain threshold, *HPT* heat pain threshold, *PHS* paradoxical heat sensation, *MDT* mechanical detection threshold, *MPT* mechanical pain threshold, *MPS* mechanical pain sensitivity, *WUR* wind up ratio, *VDT* vibration detection threshold, *PPT* pressure pain threshold.

In an exploratory analysis, we were able to detect within group differences for thermal thresholds in the intervention group, with CDT (paired *t* test, *p* = 0.002), WDT (*p* < 0.01), and TSL (*p* = 0.014). We also found gender differences for the change of TSL in the intervention group (female − 0.48 ± 0.93 °C vs. male − 0.46 ± 1.18 °C; *p* = 0.034) and for the change of WUR in the control group (female 0.01 ± 2.52 vs. male − 2.10 ± 3.56; *p* = 0.034). Sensory thresholds at baseline did not differ between genders. We did not observe side effects.

## Discussion

In this study, a homogeneous group of physically active students performed a submaximal endurance exercise and a control intervention. There were no differences in systematically assessed sensory thresholds pre- and post intervention. A 30-min long submaximal physical workload did neither result in exercise-induced hyperalgesia nor hypoalgesia.

Our data are in contrast with some previous studies, which suggested exercise-induced alterations of sensory thresholds. With regards to thermal thresholds, there are only a few studies which investigated the impact of exercise interventions. Some have proposed that exercise can mediate a change of thermal detection^21^ and thermal pain thresholds^22^. Our data (Z-profiles, Fig. 1) do not support a hypothesis of opposite trends regarding either loss or gain of function between control and exercise. All observed changes are less than one standard deviation, with changes above two standard deviations having been defined being of pathologic character^16^, and are thus of reduced clinical significance.

QST is considered the clinical gold standard in exploring pain pathologies, therefore we find the assessment of sensory profiles by applying the QST protocol as a methodological strength of our findings^15,16^. Other research groups have used different protocols, such as segmental and plurisegmental exercise-induced hypoalgesia defined as using normalized PPTs during static muscle contractions^23^. This approach is reliable to assess pressure algometry long-term^24^, and can be best understood as a comprehensive tool to assess exercise-induced hypoalgesia. Koltyn et al. assessed thermal measures besides detecting the PPT^10^. However, their approach deviated from the original QST protocol, as only contact heat evoked potentials have been applied as heat stimuli. This is known to evoke Type IV (C-fiber) temporal pain summation. The same group also focussed on molecular parameters of pain, especially the involvement of endocannabinoid markers^10,25^. Furthermore, conditioned pain modulation has been proposed to examine central pain inhibitory processing^26^. This laboratory test examines changes in sensitivity to an electrical pain stimulus applied to one part of body and after exposure to a conditioning cold pain stimulus applied to a distant part of the body. Applying this method, a recent study could not detect changes in conditioned pain modulation when comparing resistance exercisers to healthy controls^27^. Finally, our results are in line with a study applying a more refined methodology including some QST measures that could show that prolonged muscular activation (contraction) of the sternocleidomastoid muscle did not alter cold detection or mechanical thresholds^28^.

The number of other studies investigating the effects of exercise on thermal thresholds is limited. In this context, submaximal isometric knee extensions are not sufficient to alter heat pain thresholds in healthy young men^29^. Eccentric plantar flexor exercise showed no effects on the heat pain threshold in asymptomatic individuals^30^, but participants showed some reduced heat temporal summation the following morning. This could be explained by reduced central processing of painful stimuli, and confirm a previous study, which showed a trend for lumbopelvic stabilization training to increase heat but not the cold pain thresholds^31^. To our knowledge, there is no study reporting the effects of endurance exercise on thermal thresholds. One prior study has reported pain thresholds assessed in endurance athletes versus strength athletes versus controls^32^. Based on thermal threshold changes, the authors suggest that endurance-based exercise is associated with improved pain inhibition, and strength-based exercise with reduced pain sensitivity. In general, athletes have shown decreased ratings in temporal summation of pain (see above)^32^. In our study, we assessed the wind-up ratio as the mechanical correlate for temporal summation, and found no direct effects of exercise. It is therefore possible that regular exercise, but not single bouts, can affect thermal sensitivity, if at all. In this context, another point of discussion is whether the observed gender-difference in wind-up, i.e. a ratio reduced by 2 points on tenfold scale in male participants, could be a sign for a different central mechanism of exercise-induced modulation of pain perception in male and female, or an incidental finding due to multiple testing.

The number of studies investigating the effects of exercise on mechanical thresholds is more robust. Our results do neither indicate signs of exercise-induced mechanical hypo-nor hyperalgesia. To our knowledge, there is no study showing alterations of mechanical detection thresholds. Interestingly, there is one single study which showed that pre-exercise massage increased the mechanical detection threshold^33^. Both, massage and threshold, were negatively associated with muscle performance in a subsequent isokinetic exercise^33^. It is therefore important to carefully distinguish Hypo-/hyperaesthesia (detection) and Hypo-/Hyperalgesia (pain). Pain thresholds, i.e. mechanical and pressure pain, have been related extensively to the occurrence of delayed onset muscle soreness and exercise-induced muscle damage. Both thresholds are negatively affected, this corresponds to an increased pain perception^34^. Hyperalgesia in muscles is physiologically linked to the increased excitability of high-threshold mechanosensitive nociceptors, with the pressure pain threshold being the most appropriate measure^34^. All studies of this type have an eccentric muscular load in common. Thus, eccentric strength training may be categorised mediating muscular mechanical hyperalgesia. With regards to endurance (aerobic) exercise, Naugle et al. calculated a pooled moderate effect size for hypoalgesic effects on different pain thresholds^7^. The authors included two studies dealing with single mechanical thresholds (pressure), and aerobic intensities between 50 to 75% of VO_2max_. On the one hand, this is strengthened by a recent study showing aerobic exercise (cycling at 60–70% HRR for 15 min) to reduce pressure pain sensitivity but not heat pain sensitivity^35^. On the other hand, this is in contrast to another study, showing aerobic exercise until exhaustion to mediate mechanical hyperalgesia^20^. Kruger et al*.* discuss the impact of the intensity of exercise to be a key factor in a u-shaped relationship between aerobic exercise and hypo- or hyperalgesia. In summary, exercise-induced hypoalgesia may occur on a level of aerobic intensity above 75% VO_2_max with a duration of more than 10 min^20^. The chosen intensity in our study (80% HRR) is likely equal to this proposed intensity^36^, and duration of the exercise was 30 min. Thus, our results do not support the thesis that single moderate to vigorous aerobic bouts automatically lead to an alteration of the somatosensory profile.

From a clinical point of view, none of the above mentioned studies questioned if the observed changes of thresholds were of clinical relevance. In the meta-analysis by Naugle^7^, there were two studies reporting an increased pressure pain threshold following aerobic exercise. In the first study, the mean threshold increase was approximately 0.45 kg/cm^2^ in healthy volunteers^37^. The second study did not assess the exact threshold, but the time a specific weight could be withstood^38^. Kruger et al. reported a threshold decrease by approximately 8 N, which is almost 0.8 kg/cm^2^. In our study, the difference in pressure pain threshold was − 4.85 kPa (approximately − 0.04 kg/cm^2^). To explore possible clinical implications from these studies, a conversion into Z-scores is a common statistical method that re-scales the observed changes on the normative distribution within the population (see Fig. 1). Data extracted from Meeus et al. suggest a Z-score of 0.16, Krugers data from Fig. 1 can be extrapolated to a Z-score of approximately 0.4, and our study reports a Z-score of − 0.04, all based on the respective study population. All studies have in common that the conversion into Z-scores would result in threshold changes that are far from being clinically relevant.

Limitations of the present study include variations of the outdoor climate during track exercise. We carefully chose comparable climatic conditions for all study subjects, and found similar baseline thresholds at both times of assessment, but cannot completely rule out if variations in weather may have influenced the measures. In addition, the QST measure lasts approximately 30-min^16^, assessing thermal thresholds first and mechanical thresholds thereafter. To our knowledge, the duration of observed hypo- or hyperalgesic effects after exercise is not yet clear. It is therefore possible that proposed hypoalgesic effects on mechanical thresholds had already become weaned off^7^. However, the QST method has been established as the gold standard to reliably assess sustainable effects of exercise on the somatosensory system^15^. A clinically important effect size of exercise on detection and pain thresholds would have been unmasked by QST and the statistical analysis. The chosen site of measure in our study, the forehead, was selected in accordance to the Kruger study^20^, in order to make a sample size estimation and to rule out methodological differences. To our knowledge there is not yet a predefined measure site to detect sensory changes among athletes. Due to the physiology of thermoregulation^39^, it is possible that the effects of exercise at the head are more likely to affect thermal than mechanical thresholds. In addition, we found the forehead to be appropriate, as we wanted to rule out effects caused by muscular soreness and/or damage at measure sites at the extremities^34^. Therefore we cannot rule out that sensory profiles would had been different at other parts of the body, which should consequently be subject to next studies.

## Conclusion

In contrast to prior studies showing either exercise-induced hypoalgesia or hyperalgesia induced by aerobic exercise, our findings do not suggest that sub-maximal aerobic exercise alters the sensory profile at the forehead in healthy young and physically active participants. From a clinical point of view, our data suggest that previously reported sensory changes in healthy participants are of minor clinical significance. However, exercise as a therapy to treat pain in patients can be clinically meaningful. While previous studies assessed single sensory thresholds, we applied a comprehensive and standardised testing, allowing a more profound clinical analysis of descriptive findings. Further studies will be necessary to detect the impact of exercise intensity as well as the effect of exercise on other parts of the human body.
